# General Medicine and Therapeutics

**Published:** 1894-03

**Authors:** 


### GENERAL MEDICINE AND THERAPEUTICS.

EDITED BY DR. LUTHER B. GRANDY.

### THE USE OF SALOL IN DIARRHEA.

In the *Medical Chronicle* Dr. C. G. L. Skinner has a good article on this subject.

Salol is a compound of phenol and salicylic acid, containing about forty per cent. of the former and sixty per cent. of the latter. It is insoluble in water. In acid media it undergoes no change, but in alkaline fluids, and also by the action of micro-organisms, it readily splits up at the temperature of the body into phenol or carbolic acid and salicylic acid.

If then we give salol to a patient, it passes unchanged through the acid contents of the stomach, but on coming in contact with the alkaline pancreatic juice splits up into carbolic acid and salicylic acid, which thus exert their full effects on the contents of the intestines, and we have the bowel washed out with an antiseptic solution. If we take into consideration that in diarrhea absorption in the bowel is no doubt less active than in health, and also that the micro-organisms which abound in the intestines aid us in compassing their own destruction by splitting up any of the salol which may have escaped the action of the pancreatic juice, I think we must admit that, theoretically at least, salol is more apt to give good antiseptic results than the other drugs more commonly prescribed. A further advantage is that a larger dose of carbolic acid can be given in the form of salol, owing to its non-absorption in the stomach, than if the drug itself is prescribed.

May not the local action of many other drugs on the interior of the alimentary canal be too much overlooked ? Some years ago, in a paper on anemia, the late Sir Andrew Clark, after suggesting as the cause of the disease, absorption of foul gases in the intestines, gives it as his opinion that the value of iron consists, not so much in restoring the red corpuscles, as in forming an astringent lotion to apply to the interior of the bowel, thus preventing the formation of these gases. Do modern therapeutists devote too much research to dilatation and contraction of capillaries and effects on nerve endings, and too little to the immediate action of drugs on the gastro-intestinal mucous membrane?

During an epidemic of summer diarrhea, of twenty-three cases treated with salol, only one—a child eight months old—proved fatal. In very few cases were more than three or four doses necessary, and rarely were more than one or two loose stools passed after taking the first. Ordinary catarrhal diarrhea, due to errors of diet, diarrhea of children, diarrhea occurring in the course of some other disease, two or three doses seldom fail to arrest, whilst in the diarrhea of tuberculosis it can generally be relied upon to give temporary relief. It has been recommended in typhoid fever, but I have no experience of its use in that disease, nor am I aware that it has been given in cholera; but it seems to be well worth a trial, and at least as likely to prove effectual as any drug yet employed.

In all these varieties of diarrhea, the good effects of salol are most probably due entirely to its direct antiseptic action on the bowel contents—destroying bacilli, controlling acid fermentation of food and the putrefactive processes. The sedative action of carbolic acid will also lessen the peristaltic movements, and so relieve pain. The dose of salol for an adult is ten to fifteen grains (best administered in a spoonful of gruel or barley water), which may be repeated every four or six hours; to a child a year old, one or two grains may be given. It is very rarely rejected by the stomach, and in the above doses does not produce unpleasant after effects.

---

### CAMPHOR-MENTHOL IN CATARRHAL DISEASES.

Dr. Seth S. Bishop, of Chicago, in a paper thus entitled, reported a large number of cases of naso-pharyngeal catarrh, hay fever and diseases of the ear as having been treated with camphor-menthol with much better results than menthol alone produced. The presence of camphor seemed to intensify the action of menthol.

A number of hay fever sufferers, among them the president of the United States Hay Fever Association, had obtained greater relief from this inhalant than any other they had ever tried. The effect of camphor-menthol in reducing turgescence and consequent tumefaction of the turbinated bodies had rendered a comtemplated operation for stenosis unnecessary in several cases cited.

Injections of a ten per cent. solution in lanolin into constricted Eustachian tubes had caused them to become patulous. The improved ventilation of the middle ear thus effected, together with inflation with a five or ten per cent. spray of the same liquid in hypertrophic tympanic catarrh, increased the hearing and produced a sense of clearness in the head.

Cases of laryngitis, with the voices reduced to a whisper, had been treated with inhalations varying from five per cent. to twenty-five per cent. in strength, with the result of restoring the voices completely in from twenty-four to forty-eight hours.

No ill results had followed the use of this remedy in the nose, throat, larynx or middle ear. The ordinary strength of inhalation recommended by the reader was three per cent. or five per cent. for very susceptible or sensitive individuals, like hay fever patients, and ten per cent. for less nervous patients with hypertrophic catarrh, etc. In order to reduce great swelling of the turbinates and relieve stenosis, the solution shall consist of twenty or twenty-five per cent. of the camphor-menthol. The full strength of the camphor-menthol applied to eczematous eruptions relieved the itching and dissipated the redness and swelling. Similar results followed its application to hepatic eruptions.

Finally, camphor-menthol contracted the capillary blood vessels of the mucous membrane, reduced swelling, relieved pain and fullness in the head, or stenosis, arrested sneezing, checked excessive discharges and corrected perverted secretions.—*N. Y. Med. Times.*

---

### A CHALYBEATE LEMONADE.

| | | |
|---|---|---|
| ℞. | Tinci. ferri perchloridi . . . . . . . . . . . . . . | drams iv. |
| | Acidi phosphorici diluti . . . . . . . . . . . . . . | drams iv. |
| | Spiritus limonis.. . . . . . . . . . . . . . . . . . . . | drams ii. |
| | Syrupus simplicis . . . . . . ad . . . . . . . . . . | drams vi. |

Sig.—Two teaspoonfuls of the syrup in a small tumblerful of water after meals.

—*Epitome.*

